# Participation in a novel treatment component during residential substance use treatment is associated with improved outcome: a pilot study

**DOI:** 10.1186/1940-0640-9-7

**Published:** 2014-05-16

**Authors:** Kathleen P Decker, Stephanie L Peglow, Carl R Samples

**Affiliations:** 1 Hampton Veterans Affairs Medical Center, MS/18, Hampton VAMC, Hampton, VA 23667, USA; 2 Eastern Virginia Medical School, 711 Southampton Avenue, Norfolk, VA 23510, USA

**Keywords:** Person-centered care, Treatment completion, Dual diagnosis, Co-morbid, Recovery model, Residential treatment, Psychosocial treatment

## Abstract

**Background:**

A person-centered substance use treatment component, the Natural Recovery Program, was developed. The Natural Recovery Program is comprised of small group therapy combined with pursuit of hobbies.

**Methods:**

This was a pilot study of the program and was not randomized. A retrospective record review of 643 veterans in an inpatient mental health recovery and rehabilitation program was analyzed to determine if participants of Natural Recovery had a different rate of treatment completion than those who elected to participate in the core program alone. Univariate and multivariate analyses were conducted on: participation in the Natural Recovery Program; co-morbid psychiatric disorders; and legal, medical, and psychiatric issues.

**Results:**

Participation in Natural Recovery was significantly associated with successful treatment completion when analyzed by univariate analysis (p = 0.01). Other significant variables associated with successful completion included: no co-morbid psychiatric diagnosis, fewer prior suicide attempts, and no homelessness prior to admission. Binary logistic regression demonstrated that participation in Natural Recovery was associated with improved treatment completion, even when other variables were considered (p = 0.01). Treatment retention was longer for patients who participated in Natural Recovery, even if they did not complete treatment.

**Conclusions:**

The Natural Recovery Program was associated with improved outcomes, as measured by treatment retention in the first 60 days and by treatment completion. Participants of Natural Recovery with co-morbid psychiatric disorders completed treatment at a higher rate than those with co-morbid psychiatric disorders who participated in the core program. Patients reported high satisfaction with the program. This program may be a valuable adjunct to residential treatment.

## Background

Residential treatment completion has been shown to be an important predictor of long-term positive outcomes, including both improved abstinence [1] and reduced re-admission [2]. Patients who complete residential treatment benefit from decreased substance use, fewer legal problems or risky behaviors [3-6], lowered mortality [7], more employment [3,5], and reduced suicide rates [8]. Some of the known factors associated with residential substance use treatment completion are summarized below.

The Treatment Episode Data Set (TEDS), an ongoing national survey of admissions and discharges for substance use treatment, showed that the following factors were associated with a higher rate of treatment completion: more than high school education, Caucasian ethnicity, history of employment prior to admission, older age, less frequent use of substances, and male gender [9]. Other investigators have shown that factors such as childhood neglect, higher severity of use, more intense treatment history [10,11], and more arrests prior to treatment [12] all are predictors of poor completion rates and often are associated with poor long-term outcomes.

More severe Axis I psychiatric co-morbidity has been associated with lower completion rates in many studies of residential substance treatment [13-15]. Specifically, more severe anxiety [16] and mood instability [13] are associated with lower completion rates. The presence of drug or alcohol craving [10,14,17] also is associated with a lower completion rate. Programs that encourage treatment of dual disorders improve outcomes in patients with dual diagnosis [18,19].

Patients with Axis II co-morbidity, including Antisocial Personality Disorder (ASPD), benefit from substance use treatment. Previous studies show that patients with ASPD randomized to an abbreviated (versus standard) alcohol inpatient treatment program combined with outpatient treatment demonstrated that patients with ASPD were as likely to complete treatment as patients without ASPD. They exhibited the same patterns of reduced drug use and recidivism as did patients without ASPD [20]. Court-mandated drug treatment has been associated with higher treatment completion rates for some patients with ASPD [10].

Patient satisfaction with treatment is associated with better treatment outcomes. In one study, greater service intensity and satisfaction were positively related to treatment completion and longer treatment retention, which in turn related to more favorable outcomes. In that study, patients with greater initial problem severity received more services and were more likely to be satisfied in both outpatient and residential drug treatment programs [4]. Women in residential treatment who endorsed beliefs, including control over their health status, and perceived their sponsor as helpful in Alcoholics Anonymous (AA) were more likely to complete treatment [21].

Long-term follow-up of patients with substance use disorders shows that 12-step programs for substance use dependence have had significant effects on outcome and relapse rate [22]. Also, professionally designed programs that incorporate 12-step components have better treatment completion rates and lower relapse rates in patients at follow-up than programs which lack 12-step components [23-25]. Finally, adherence to 12-step aftercare programs following residential treatment completion improves long-term outcomes [26]. The principal author (KD) theorized that one major advantage of 12-step programs over professional programs is the absence of (perceived) value judgments or (perceived) stigma that may result in patients who are lectured about substance use disorders by professionals. Recent studies suggest that confrontation about substance use disorders is perceived more positively when it is less directive and delivered by trusted figures [27,28]. Additionally, individuals who have difficulty with authority figures, such as those with ASPD traits or Cluster B spectrum traits, may resist formal classes and “education” compared to receiving information or discussing addiction with peers. Hence, delivery of psychoeducational and therapeutic material in Natural Recovery is by peers who read the modules aloud, then discuss them as a group. A substance use professional facilitates the discussion (as opposed to lecturing) and allows the peer group to give most of the feedback to participants.

The Natural Recovery Program also addresses Axis II characteristics common to substance use disorder and ASPD such as impulsivity, risky behavior, lack of concern for consequences to self or others; these problems are addressed by analogy to learning new hobbies or engaging in preferred hobbies. For example, instead of lecturing participants on the dangers of impulsivity and risky behaviors, the module on impulsivity begins with a discussion of how planning can result in better music performance (Natural Recovery-Art/Music) or better growth of a gardener’s plants (Natural Recovery-Horticulture). This allows participants to internalize the benefits of planning by analogy to their hobby, a pleasurable topic. Then, as participants discuss past impulsive behaviors, they may feel freer to examine past impulsive behaviors without feeling stigmatized or judged negatively.

The Natural Recovery Program utilizes elements of motivational enhancement, cognitive-behavioral treatment, and psychoeducational material in an interactive style, with professional facilitation in a person-centered manner. Although investigators use the term “person-centered care” to mean different things, most agree that key concepts include: re-orientation from patient to personhood; re-definition of valued knowledge and expertise; and most importantly, a partnership and negotiation in decision-making between client and provider [29,30]. Natural Recovery was designed so that professionals with a wide range of mental health training could facilitate it, including health technicians, addiction therapists, as well as psychologists or psychiatrists.

Another feature of the program design was that by engaging patients in hobbies while discussing substance use issues, patients would associate pleasurable activities with the recovery process, rather than aversion or punishment. This might enhance motivation to change and retention in the program and thus improve outcome.

Another goal of the Natural Recovery Program is to teach patients to engage in hobbies instead of using substances on weekends, and to re-experience (or begin to experience) pleasure from hobbies instead of substances of abuse. Instead of lecturing patients to use leisure time in healthier ways, patients practice leisure activities, while sober, for weeks at a time in a structured setting and thus acquire confidence that they can engage in pleasurable activities without using substances after discharge. Since the mean lifetime “clean and sober” interval of patients in the Drug Abuse Program (RRTP) is less than 2 years prior to treatment, this population of patients with severe substance use dependence has little experience or confidence that they can engage in pleasurable activities without substance use on admission (Stack, Samples, Decker, unpublished observations, 2014).

The format of the Natural Recovery Program is 1-hour group therapy sessions during the workweek using therapy modules (14 total sessions), followed by pursuit of a preferred hobby for 4 hours a day on weekends. Participants may spend 4 hours either individually or with other group members working on the hobby. Multiple tracks have been designed to accommodate different hobbies, but the modules cover the same concepts. Analogies between substance use treatment concepts and hobbies are constructed specifically for each track. For example, the module of competition in Natural Recovery-Art/Music track uses the analogy of art and music competitions, whereas the Natural Recovery-Horticulture track uses the analogy of weeds competing with seedlings for survival. In each track, an analogy is made between competition as illustrated in the chosen hobby to competition for love or attention as a child and the consequences of these needs not being met. The exercise accompanying the educational material is also the same for both tracks; in this module, participants discuss how competition played a role in their development before and after developing substance dependence.

Humans utilize analogical reasoning to facilitate learning new information, which improves learning compared to procedural instruction [31]. Several sub-regions of prefrontal cortex have been shown to be specifically engaged in concrete and abstract analogical learning, as well as visuospatial learning [32]. Studies in humans and in rhesus monkeys have shown that chronic alcohol use leads to defects in learning and memory in these brain regions [33,34]. Cocaine dependence also has been shown to be associated with defective learning and pre-frontal cortical deficits [35]. The principal author (KD) hypothesized that presenting analogies verbally between preferred hobbies and concepts involved in recovery from substance use disorders might enhance internalization of psycho-educational material by facilitating analogical learning.

This study reports data from the two initial Natural Recovery tracks-Art/Music and Horticulture. The goal of this pilot study was to compare treatment retention, treatment completion, and satisfaction of patients who participated in the Natural Recovery-Horticulture track or Natural Recovery-Art/Music track versus patients in the same substance use program who received the same number of hours of treatment with psychoeducational lectures (RRTP core program, or RRTP-CP).

### Participants

The records of 643 veterans treated in a RRTP residential rehabilitation treatment program at a Veteran’s Administration hospital between November 2009 and March 2011 were analyzed retrospectively in a quasi-experimental design. The researchers were not blind to participation, and participation in the program was an elective component, not randomized. All patients enrolled in RRTP were eligible to participate in a novel program called Natural Recovery. Fifteen percent participated in Natural Recovery-Horticulture (n = 101), 5 percent participated in Natural Recovery-Art/Music (n = 30) and 79 percent elected to participate in the RRTP-CP only RRTP (n = 512). One percent (n = 8) participated in both tracks of Natural Recovery. Univariate analysis was performed to characterize similarities and differences between those who elected to participate in Natural Recovery-Art/Music versus Horticulture versus RRTP-CP. Forty participants of RRTP-CP were re-admitted to RRTP during the study (8%). Two participants of Natural Recovery-Horticulture were re-admitted to RRTP during the study, which was significantly lower compared to RRTP-CP (2%; p < 0.01). Three participants of Natural Recovery-Art/Music were re-admitted to RRTP (10%), which was not significantly different compared to RRTP-CP. For this study, each admission of a participant was treated as a separate event.

### Procedures

All study procedures were reviewed and approved by the Hampton Veterans Affairs Medical Center Institutional Review Board. As this was a retrospective records review, an informed consent waiver was obtained.

Participants in Natural Recovery received a 1-hour small group therapy session during the week using modules with a staff facilitator, while RRTP-CP participants attended a large psychoeducational group at that time. Participants in Natural Recovery were given a choice of tracks, either Horticulture or Art/Music, based on personal preference. The modules employ analogies between the recovery process and participants’ chosen hobby. Although hobbies were different between the two tracks of Natural Recovery, the 14 modules covered the same concepts, while only the analogy portion of each handout differed.

Natural Recovery participants also pursued 4 hours of their hobby on each weekend day, while RRTP-CP participants received large-group psychoeducational lectures on weekends for the same amount of time. Thus, total substance use treatment hours between Natural Recovery participants and RRTP-CP participants were equivalent. Additionally, all Natural Recovery-Art/Music, Natural Recovery-Horticulture, and RRTP-CP participants received one “leisure education” lecture.

All participants were free to pursue hobbies during treatment, but Natural Recovery participants received materials (access to gardening tools, gardening zones, and musical instruments and/or art supplies) to pursue their choice of hobby on the weekend. RRTP-CP participants could pursue hobbies during treatment but were not provided musical instruments or art materials, nor did they have access to gardening tools to work in the garden, although they could walk in it.

## Materials

The modules of Natural Recovery previously have been published and constitute 14 topics, each of which is discussed weekly for 14 weeks [36]. Participants could repeat Natural Recovery modules if their length of stay exceeded 14 weeks. The total number of modules was recorded for each participant.

### Statistical methods

Demographic information including age, gender, and educational level was collected. Homelessness at time of admission, chronic pain, dental problems, prior substance use treatment, legal history, and substance preference was collected, as was the number of days in treatment, co-morbid Axis I and II disorders, and military service time and era. Prior substance use treatment was recorded as the number of prior intensive outpatient treatment episodes and the number of prior inpatient treatment episodes. Patient satisfaction was measured at time of discharge by patient ratings of 1–5, where 5 represented highest satisfaction with each of a number of program components, including participation in Natural Recovery and RRTP-CP.

These variables were analyzed to determine factors associated with higher treatment retention or successful completion. Univariate analysis (chi-square or t-test) was conducted to determine characteristics that differed between individuals who completed treatment: Axis I disorders, Axis II disorders, prior suicide attempts, homelessness on admission, legal history, sexual abuse as a child, back pain, marital status, time in service, service era, the number of prior inpatient substance use treatment episodes, the number of intensive outpatient substance use treatment episodes, dental issues, and participation in Natural Recovery. Univariate analyses as well as binary logistic regression analyses were conducted to control for significant variables and to analyze the contribution of each independent variable to the overall predictive model. Statistical analysis was conducted using SPSS, version PASW 18.

### Missing data

Irregular discharge was defined as failure to complete treatment due to either: being absent from the program without leave, staff request, relapse during the program, or discharge against medical advice. Patients who were irregularly discharged had missing information for patient satisfaction with Natural Recovery or RRTP-CP, as they did not properly complete paperwork at the time of discharge. Other data, including demographic, diagnostic, medical, or legal history, were not missing because they were collected at admission.

## Results

### Demographics

There were some demographic differences between participants in Natural Recovery tracks and RRTP-CP, as shown in Table 1. Differences included: a higher percentage of Caucasian participants and a lower percentage of African American participants in both tracks of Natural Recovery compared to RRTP-CP (36.6%, 57.4% in Natural Recovery-Horticulture and 53.3%, 46.7% in Natural Recovery-Art/Music versus 25.4%, 73.4% in RRTP-CP; p < 0.01); fewer married participants than in RRTP-CP (5% in Natural Recovery-Horticulture and 7% in Natural Recovery-Art/Music versus 15% in RRTP-CP; p < 0.01); more divorced participants (48% in Natural Recovery-Horticulture and 67% in Natural Recovery-Art/Music versus 45% in RRTP-CP; p < 0.01). There were more homeless participants in RRTP-Natural Recovery-Horticulture and Natural Recovery-Art/Music (67% and 63%, respectively, versus 60% in RRTP-CP; p < 0.001) and fewer veterans of Operation Iraqi Freedom/Operation Enduring Freedom (5% in Natural Recovery-Horticulture and 3% for Natural Recovery-Art/Music versus 7% for RRTP-CP; p < 0.01). There was no significant difference in participation by gender and no significant difference in mean age (50.7) or years of education (12.4) between RRTP-CP and either track of Natural Recovery.

**Table 1:** Demographic information of participants in Natural Recovery Horticulture track (NR HORT) or Art/music (NR ART/MU) vs. RRTP-core program (RRTP-CP)

**ETHNICITY**	**RRTP-CP (n = 512)**	**NR HORT (n = 101)**	**NR ART/MU (n = 30)**
African American	73.4%	57.4%*	46.7%*
Caucasian	25.4%	36.6%*	53.3%*
Hispanic	0.6%	1.0%	0
Hawaiian Islander	0.2%	0	0
Native American	0	2.0%	0
**MARITAL STATUS**			
Divorced	45%*	48%*	67%*
Married	15%*	5%*	7%*
Separated	17%*	15%*	10%*
Single	20%*	26%*	17%*
Widowed	3%	4%	0%
**Age** (years)	50.7	49.7	44.8
**Years of Education**	12.4	12.4	12.7

*p < 0.01.

More participants of both tracks of Natural Recovery had a primary diagnosis of alcohol dependence (50% of Natural Recovery-Horticulture and 57% for Natural Recovery-Art/Music versus 43% for RRTP-CP; p < 0.01). Fewer participants of both tracks of Natural Recovery had a primary diagnosis of cocaine dependence (32% of Natural Recovery-Horticulture and 27% of Natural Recovery-Art/Music versus 45% of RRTP-CP; p < 0.01). The percentage of participants with opiate dependence was not significantly different between either track of Natural Recovery compared to RRTP-CP (14% for Natural Recovery-Horticulture, 10% for Natural Recovery-Art/Music versus 10% for RRTP-CP). In addition, there was no statistically significant difference between the percentages of participants in either track of Natural Recovery who completed treatment by primary substance dependence.

### Treatment retention and completion

RRTP is a 60-120–day residential substance use treatment program divided into two phases. In Phase I, which typically lasts 60 days, all individuals are given a combination of intensive substance use treatment offerings including psycho-educational groups, several hours a week of small group therapy, and individual case management sessions as needed. Individuals who remain for Phase II pursue competitive employment. They attend only 1 hour of substance use group therapy a week and also have 1 hour of individual therapy per week. Most of their time in Phase II is spent working at Compensated Work Therapy jobs and searching for competitive community employment and housing.

**Figure 1:**
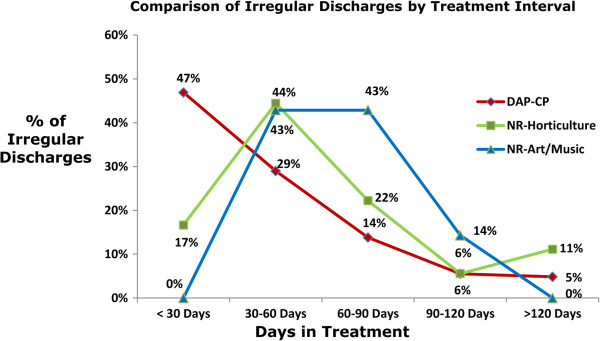
**Comparison of Irregular Discharges by Treatment Interval.** Comparison of percentage of irregular discharges in each of three tracks by treatment interval. The percentage of irregular discharges by treatment interval is displayed for RRTP-CP, Natural Recovery-Horticulture and Natural Recovery-Art/Music. Each data point represents the percentage of all irregular discharges for that track at the specified treatment interval.

Treatment retention, as measured by length of stay, was compared between participants of Natural Recovery and those in RRTP-CP. Participants who completed two or more modules of Natural Recovery-Horticulture had a statistically significantly greater mean length of stay (89 versus 74 days; p < 0.01). Participants who completed two or more modules of the Natural Recovery-Art/Music track also had a statistically significantly greater mean length of stay (90 versus 74 days; p = 0.03).Results also demonstrate that 76% of the irregular discharges from RRTP-CP occurred in the first 60 days of treatment, or Phase I, during which intensive substance use treatment offerings occur. In contrast, only 61% of irregularly discharged participants from Natural Recovery-Horticulture and 43% of irregularly discharged participants in Natural Recovery-Art/Music occurred within the first 60 days of treatment, as shown in Figure 1 (p < 0.01). One participant of Natural Recovery-Art/Music and zero participants of Natural Recovery-Horticulture were irregularly discharged within the first 30 days, but these findings did not achieve statistical significance due to small numbers. Thus, even the Natural Recovery participants who were irregularly discharged received more of the early intensive substance use treatment programming than those irregularly discharged from RRTP-CP.

Treatment completion was defined as completing the program with a regular discharge, whether the participant completed Phase I only or remained through Phase II. Univariate analysis demonstrated that participation in Natural Recovery was significantly associated with successful treatment completion. Participants of Natural Recovery-Horticulture completed treatment at a higher rate overall than participants of RRTP-CP in univariate analysis (82% versus 72%; p = 0.05). More participants of Natural Recovery-Art/Music completed treatment than RRTP-CP (77% versus 73%), but the difference was not statistically significant.

Binary logistic regression analysis was conducted with treatment completion as the binary outcome variable. Participation in the Natural Recovery-Horticulture track was significantly associated with treatment completion when other variables associated with successful completion were included (p = 0.01). These variables included: male gender, African American ethnicity, lack of co-morbid Axis I disorder, fewer felony convictions, lack of dental problems, and fewer prior suicide attempts (Table 2). These variables also were associated with successful treatment completion in an outcome study conducted in the same program in the same facility a decade prior to this study, with the exception of legal history [37]. In the prior study, legal history was not separated by felony versus non-felony convictions and was not significantly associated with treatment completion.

**Table 2:** Binary logistic regression analysis of factors associated with treatment completion

	**Sig.**	**OR**		**95% C.I. for OR**
			**Lower**	**Upper**
Natural Recovery-Horticulture	.01	2.12	1.18	3.80
Natural Recovery-Art/Music	.20	1.77	.74	4.23
Female Gender	.04	.52	.27	.98
**Ethnicity**	.50			
African American	.05	1.53	1.00	2.34
Hispanic	.87	1.22	.12	12.42
Hawaiian Islander	1.00	.00	.00	
Native American	.64	.54	.04	6.84
Caucasian	1.00	.00	.00	
Prior Suicide Attempts	.04	.87	.76	1.00
Dental Problems	.04	.59	.36	.98
Co-morbid Axis I Disorder	.01	.59	.39	.89
Co-morbid Axis II Disorder	.17	.71	.44	1.15
Felony Conviction	.01	.88	.08	.97
Non-felony Conviction	.12	.96	.91	1.01
Age	.95	1.00	.98	1.02
**Marital Status**	.65			
Divorced	.66	1.26	.45	3.53
Married	.30	1.82	.58	5.72
Separated	.43	1.55	.52	4.66
Single	.39	1.62	.55	4.79
Homeless Prior to Admission	.26	.80	.54	1.18
Prior Intensive Outpatient Substance Use Treatment Episodes	.45	1.08	.89	1.31
Prior Inpatient Substance Use Treatment Episodes	.93	1.00	.92	1.09
Court-Ordered to Treatment	.33	.50	.13	2.01
Back Pain	.52	1.14	.78	1.66
Constant	.06	8.23		

Participation in Natural Recovery-Art/Music was not significantly associated with treatment completion in binary logistic regression analysis. Other variables that were not significantly associated with successful treatment completion in this study included: marital status, age, back pain, court-ordered status, the number of prior substance use treatment episodes, or homelessness prior to admission.

### Co-morbid psychiatric disorders

Table 3 shows the proportion of participants with co-morbid Axis I disorders in Natural Recovery-Horticulture was higher, but not statistically different, than those in RRTP-CP with co-morbid Axis I disorders (63% versus 54%). Participants with combined co-morbid Axis I disorders who participated in Natural Recovery-Horticulture had a significantly higher treatment completion rate compared to those with combined co-morbid Axis I disorders in RRTP-CP (79% versus 65%; p = 0.02). The proportion of participants with no co-morbid Axis I disorder who completed treatment in the Natural Recovery-Horticulture track was higher, but not significantly higher, than those with no co-morbid Axis I disorder in RRTP-CP (87% versus 80%).

**Table 3:** Treatment completion by co-morbid Axis I Disorder

	**RRTP-CP**		**NR-Horticulture**			**NR-Art/Music**		
	**Total Participants (n)**	**Completed Treatment %**	**Total Participants (n)**	**Completed Treatment %**	**p-value**	**Total Participants (n)**	**Completed Treatment %**	**p-value**
Substance-Induced Disorders	12	75	0	n/a	n/a	0	n/a	n/a
Dementia, Cognitive Disorder	3	100	0	n/a	n/a	0	n/a	n/a
Schizophrenia, Schizoaffective	15	60	3	100	0.00	3	100	0.18
Major Depression	37	68	16	81	0.31	3	100	0.24
Bipolar I, II	31	58	9	67	0.64	5	80	0.35
Bipolar NOS & Mood Disorder NOS	23	61	3	67	0.36	4	100	0.13
Malingering	3	100	2	100	n/a	1	100	
Anxiety Disorders	14	64	4	75	0.69	1	100	0.47
Dysthymic Disorder	21	52	3	67	0.54	1	100	0.35
Depression NOS	102	71	17	81	0.00	9	55	0.35
Attention Deficit Disorder	4	50	4	100	0.10	1	100	0.36
Adjustment Disorder	7	29	2	100	0.07	0	n/a	n/a
All Other Axis I Diagnoses	5	60	0	n/a	n/a	0	n/a	n/a
**Total with co-morbid disorders**	277	65	63	79	0.02	25	72	0.49
**Total with no co-morbid disorder**	235	80	37	87	0.35	5	80	1.00

Statistical conclusions with respect to individual co-morbid disorders were limited due to small sample size. More participants in Natural Recovery-Horticulture with depression not otherwise specified (NOS) completed treatment, compared to participants with depression NOS in RRTP-CP (81% versus 71%; p < 0.01). More participants of Natural Recovery-Horticulture with major depression completed treatment (81%) than participants with major depression in RRTP-CP (68%); and more participants in Natural Recovery-Horticulture with bipolar disorder I or II completed treatment (67%) compared to bipolar participants enrolled in RRTP-CP (58%), but neither of these findings achieved statistical significance.

The proportion of participants with co-morbid Axis I disorders in Natural Recovery-Art/Music was significantly higher compared to RRTP-CP participants with co-morbid Axis I disorders (83% versus 54%; p < 0.01). There was a trend to higher completion rate in those with combined co-morbid Axis I disorders in Natural Recovery-Art/Music compared to RRTP-CP, but it was not statistically significant (72% versus 65%, respectively), as shown in Table 3. There were no statistically significant findings for individual disorders due to small sample size.

Participants of RRTP-CP with Axis II disorders had a lower treatment completion rate compared to participants who did not have an Axis II disorder (53% versus 77%; p < 0.01). Participants of Natural Recovery-Horticulture with an Axis II disorder completed treatment at a higher rate than RRTP-CP participants with Axis II disorders (89% versus 53%RRTP; p < 0.01). More patients with ASPD who participated in Natural Recovery-Horticulture completed treatment than those with ASPD in RRTP-CP (91% versus 54%; p = 0.02). The number of participants in Natural Recovery-Horticulture with other Axis II diagnoses was too small to achieve statistical significance.

Participants of Natural Recovery-Art/Music with Axis II diagnoses also completed treatment at a higher rate compared to RRTP-CP participants with Axis II disorders (100% versus 53%, respectively), but results did not achieve statistical significance due to small sample size. Two participants with Cluster A disorders had a 100% completion rate; one was a participant of RRTP-CP and one was a participant in Natural Recovery-Art/Music.

### Patient satisfaction

Satisfaction was tracked for two reasons. The Natural Recovery Program was designed to be a person-centered, recovery-oriented program, and patient satisfaction is key with such programs. The second reason that satisfaction was viewed as important is because the Natural Recovery Program was originally studied as a performance improvement measure, to ensure that patients were no less satisfied than those in RRTP-CP.

The mean rating for patient satisfaction at the time of discharge with the Natural Recovery Program for each track was 4.5 on a 5-point scale, where 5 was highest. Satisfaction with RRTP-CP was not statistically different from that of either Natural Recovery track (4.6).

Specific comments from patients about the Natural Recovery Program included:

“When I leave RRTP there is something I can get involved in to forget about my addiction.”

“To focus on recovery while enjoying myself at the same time has been extremely therapeutic.”

“Natural Recovery is rewarding!”

“Being able to play musical instruments during weekends allows me to think more with my spirit than my mind.”

## Discussion

One improved outcome of substance use rehabilitation in participants of the Natural Recovery Program compared to RRTP-CP was a lower irregular discharge rate in early treatment. The first 60 days of RRTP is the most intensive portion of the program with respect to substance use treatment. In a prior study on patients pooled from Veterans Health Administration substance use rehabilitation programs, retention early in treatment (45 days or less) was associated with an improved outcome as measured by a self-administered Addiction Severity Index (ASI) completed 6 months after treatment completion. Programs with length of stay longer than 90 days had no further significant improvement in ASI [38]. Therefore, the authors hypothesized that retention early in substance use rehabilitation programs is critical because the intensive substance use offerings are concentrated early in treatment, whereas retention after 90 days is less important to substance use outcome.

In a study on a civilian community population with higher-functioning patients and different demographic composition than this one, longer length of stay in residential treatment did not lead to improved abstinence [39]. However, in that study patients had an index hospitalization of up to 30 days prior to residential treatment, and length of stay up to 30 days in the index hospitalization was a predictor of positive outcomes [39]. Therefore, both these studies appear to indicate that length of stay in early treatment (between 30 to 90 days) is associated with improved substance use outcomes, but that length of stay longer than 90 days may not be associated with improved substance use outcomes. Thus, the fact that more participants of Natural Recovery were retained in treatment within the first 30–60 days may represent an improved outcome.

Overall trends were similar in terms of both improved treatment retention and completion with both Natural Recovery tracks compared to RRTP-CP, but the data was statistically different for Natural Recovery-Horticulture compared to RRTP-CP. It is difficult to determine what caused the improved outcomes of Natural Recovery-Horticulture compared to RRTP-CP, whereas the lack of significantly improved outcome of Natural Recovery-Art/Music with respect to treatment completion is due to differences in participant attributes or due to the small sample size of Natural Recovery-Art/Music. A follow-up study with more participants is planned.

There were some differences in participant attributes between Natural Recovery-Art/Music, Natural Recovery Horticulture, and RRTP-CP. In addition to the higher percentage of participants with co-morbid Axis I disorders in Natural Recovery-Art/Music, there were more Caucasians in the Natural Recovery-Art/Music track compared to RRTP-CP or Natural Recovery-Horticulture in this study (53%, 25%, 37%, respectively; p < 0.01). Caucasians completed treatment at a lower rate. Natural Recovery-Art/Music had significantly more divorced participants than either RRTP-CP or Natural Recovery-Horticulture (67%, 48%, 45%, respectively; p < 0.01). Although logistic regression analysis took into account these factors, there may be other differences in participant attributes, such as severity of illness or differences in impulse control, which resulted in different outcomes or interactions of multiple factors associated with lower treatment completion.

Both tracks of Natural Recovery had more participants with alcohol dependence as their primary diagnosis compared to RRTP-CP. The significance of this is unclear, as primary substance use diagnosis was not related to treatment completion in the logistic regression analysis.

More participants of Natural Recovery-Art/Music had co-morbid Axis I disorders than Natural Recovery-Horticulture, and although the proportion of participants with Axis I disorders was not significantly different between Natural Recovery-Horticulture and RRTP-CP, individuals in both tracks of Natural Recovery with co-morbid disorders completed treatment at a higher rate. This suggests that Natural Recovery may be a useful adjunctive treatment for individuals with co-morbid disorders.

The finding that participants in Natural Recovery enjoyed the program and found it “rewarding” and “enjoyable” is encouraging, and it may explain why participants of Natural Recovery were less likely to be irregularly discharged from the program early in treatment. The Natural Recovery Program was developed to address important substance use treatment issues and Axis II issues related to antisocial behavior in a person-centered manner that was enjoyable and non-stigmatizing, so it appears that it was successful in this pilot study, both in terms of patient satisfaction and treatment completion. Many of the participants’ comments specifically addressed their satisfaction with the active practice of hobbies, as opposed to lecture format.

The Natural Recovery Program is a low-cost program to administer. The supplies for each track can be obtained for less than $200/month. Groups are designed so that a wide range of facilitators, including occupational therapists, addiction therapists and art or music therapists, as well as licensed social workers, psychologists, or psychiatrists can administer them. A trial is planned with peer counselors running the program as well.

Transference and counter-transference issues with patients provided both unanticipated benefits and challenges. Patients may have responded more positively to Natural Recovery facilitators because they were able to demonstrate and/or cultivate their strengths to staff in terms of hobbies. This positive relationship may have contributed to participants’ retention and completion of treatment. In prior research, a survey of recovering substance users indicated that support and encouragement during substance use treatment by healthcare providers was helpful in recovery, but negative attitudes and/or a negative relationship with providers led patients to conceal their problems [28]. Additionally, poor treatment alliance has been associated with a higher dropout rate from substance use treatment [40]. Future work may address participants’ perceptions of their relationships with staff. At times, participants also expected “special treatment” or viewed staff as peers due to the non-authoritarian approach by facilitators and the patients’ expertise in their hobbies. Therefore, attention to professional boundaries is important, in spite of the enjoyable treatment activities and positive relationships with staff.

Participants in Natural Recovery rated the program very highly in terms of patient satisfaction. Therefore, Natural Recovery participation may increase patient satisfaction with treatment, leading to improved treatment completion [4]. This program may be a valuable component in residential treatment, especially for dual diagnosis patients. These findings will be tested in a randomized, controlled trial to further determine if the Natural Recovery Program is a useful program component in substance use treatment programs. A manual of the program has been published [36] and also is freely available from the principle author. Additionally, the author has developed a DVD with a video of two simulated 1-hour Natural Recovery sessions for teaching purposes for investigators or programs that are interested in assisting with dissemination of the program.

Limitations of the study included that it was not a randomized study and therefore, there was no control for motivation for change. Although logistic regression suggests that co-morbid disorders (other than ASPD) do not account for different treatment completion rates, some individuals may have the same disorder but be in different stages of motivation for treatment. In other words, perhaps individuals with co-morbid disorders who elected to participate in Natural Recovery were more motivated for change and therefore completed treatment at a higher rate. However, in multi-site trials using one of the most widely used measures of motivation, stated motivation to change was not significantly associated with differences in treatment outcome, although the measure was statistically robust [41].

Another possibility to explain the improved outcome is that participants of Natural Recovery developed a more positive relationship with staff as a result of person-centered treatment using hobbies. This suggests an avenue for further study: assessing not only the motivational state of participants prospectively, but analyzing participants’ perceptions of their relationships with staff after participating in this person-centered treatment program.

Other limitations include that there were few female veterans, few veterans of Operation Iraqi Freedom/Enduring Freedom, and few Hispanic or Asian patients in the study population. Therefore, it is unclear to what degree these findings can be generalized to other populations. Although patients participating in Natural Recovery completed treatment at a higher rate, it is also unknown whether this outcome will be related to sustained improvement. Finally, patients who left the program prematurely or were irregularly discharged did not complete feedback on the program and, given that they represent a high-risk category, excluding their input on satisfaction with the program, while necessary, was unfortunate.

## Conclusions

The Natural Recovery Program was associated with improved outcome as measured by higher rate of treatment retention in the first 60 days as well as during all stages of treatment, a higher rate of treatment completion, and high patient satisfaction. Participants of Natural Recovery with co-morbid psychiatric disorders completed treatment at a higher rate than those with co-morbid disorders who did not participate in it. This program may be a valuable adjunct to residential treatment, especially in the early stages of substance use disorder treatment, when retention is associated with improved long-term outcomes for substance use disorders. There is an urgent need for both improved outcomes as well as recovery-oriented treatment with greater patient satisfaction. In addition, treatment that can be delivered in group format to accommodate the burgeoning veteran population with mental health and substance use treatment needs is preferable where possible.

## Competing interests

The authors declare they have no ethical or competing interests.

## Authors’ contributions

KD designed the novel treatment program and conceived of the study, conducted the statistical analyses, and drafted the manuscript. SP conducted portions of the literature search and writing. CS acquired and maintained data. All authors participated in revising the manuscript critically for specific important intellectual content and have given final approval of the version to be published.
